# Hyoscine butylbromide induced psychosis: A case report

**DOI:** 10.1002/ccr3.5807

**Published:** 2022-05-05

**Authors:** Nimesh Lageju, Durga Neupane, Lokesh Shekher Jaiswal, Upama Phuyal

**Affiliations:** ^1^ MBBS B.P. Koirala Institute of Health Sciences Dharan Nepal; ^2^ Department of Surgery (Division of CTVS) B.P. Koirala Institute of Health Sciences Dharan Nepal; ^3^ 12329 The University of Texas at Arlington Arlington Texas USA

**Keywords:** hallucination, hyoscine butylbromide, physostigmine, psychosis

## Abstract

Hyoscine butylbromide–induced pyschosis, though rare, should be considered in a child presenting with deteriorating cognitive functions and psychotic features acutely as evident in our case of a 9‐year‐old child taking hyoscine for her non‐specific abdominal pain.

## Abstract 2

Through this case, we hope to communicate the notion that every practitioner should be aware of common medicines with anticholinergic effects, and drug‐induced psychosis should be investigated in young patients with acute behavioral abnormalities.

## 1 INTRODUCTION

Hyoscine is a non‐selective muscarinic acetylcholine receptor antagonist that binds to a group of muscarinic receptors (M1‐M5) that modulates the release of acetylcholine or dopamine indirectly. M1‐like muscarinic receptors (M1, M3, and M5) and M2‐like muscarinic receptors (M2 and M4) activate and inhibit G protein‐mediated second messenger transduction, respectively.^1^, ^2^ Hyoscine blockade of the M2 muscarinic auto‐receptor in the axon terminal is thought to raise acetylcholine levels in the substantia nigra (SN) and ventral tegmental area (VTA) midbrain areas, increasing striatal dopaminergic levels in the mesopontine nuclei.^3^, ^4^ Positive psychotic symptoms such as hyperactivity and stereotypy may result from such activity.^5^, ^6^ Inhibition of M4 subtype receptors, on the contrary, has been linked to memory and attention problems.^1^We report a case of hyoscine‐induced psychosis in a 9‐year‐old female patient. The psychosis began when the medicine was taken and faded once it was stopped. After a few days of being off the medicine, the child was perfectly fine.

## 2 CASE PRESENTATION

A 9‐year‐old female patient was presented to the emergency room of our hospital with complaints of exhibiting abnormal behaviors. The symptoms had developed over the previous six hours. She complained of being dizzy and experiencing a dry mouth. Intermittent hallucinations started. She described her visions of “her friends playing games.” She heard “her mother's voice calling her.” She also had a tactile hallucination in which she felt something crawling on her back. She constantly attempted to pick imaginary things from the floor.

Upon arrival at the emergency, the girl was hyperactive and was held still by her uncle. She had a blood pressure of 110/70 mm of Hg, a heart rate of 92/min, a temperature of 98.2 ⁰F, and a respiratory rate of 20/min. Her mouth and skin were dry. Pupils were slightly dilated and reactive to light. She was constantly agitated and restless. Her cognitive deficits were quite remarkable because she was unable to recognize previously familiar people and was not oriented to place or time. There were no focal neurological abnormalities found. There was no history of head trauma, and no obvious injuries were discovered. A day prior, the girl had visited outpatient department (OPD) for complaints of abdominal pain for which hyoscine butylbromide 20 mg was prescribed to be taken three times a day.

Laboratory results for hemoglobin, total leukocyte count, platelet count, urine routine and microscopy, liver function test, renal function test, and random blood sugar were within normal limits. The chest X‐ray was normal. Due to her bizarre behavior and suspicion of hyoscine intoxication, she was admitted for further evaluation. A pediatrician and a psychiatrist were consulted. The prescription of hyoscine was stopped.

The evening after admission, the girl showed improvement in her symptoms including her memory and hallucinations. She was no longer hyperactive and began to become shy. After proper counseling to her relatives, she was discharged. On follow‐up, there was no recurrence of those symptoms and she was completely recovered.

## 3 DISCUSSION

This case exemplifies several issues that can arise when diagnosing toxic psychosis caused by orally administered hyoscine. Hyoscine is usually prescribed in adult patients for the treatment of abdominal pain associated with cramps induced by gastrointestinal (GI) spasms. It is an antimuscarinic medication that works similarly to atropine. It is made from a belladonna alkaloid (Atropa belladonna, commonly known as deadly nightshade). It antagonizes acetylcholine at muscarinic receptors at therapeutic doses, but it can also antagonize nicotinic receptors at high doses.^7^

Confusion, hallucination, fatigue, short‐term memory loss, difficulty urinating, blurred vision, skin redness, and changes in heart rate are all common complications or side effects of hyoscine butylbromide.^8^ A person suffering from anticholinergic intoxication can be described as "red as a beet, hot as a hare, dry as a bone, blind as a bat, mad as a hatter." This is a representation of a patient's flushed skin, hyperthermia, dry mucous membranes, blurred vision, and confusion or delirium.^9^ Most cases of hyoscine intoxication can be treated by stopping the offending drug and providing supportive care to lower the patient's fever, keep them cool, and keep them hydrated. Benzodiazepines can be used to reduce agitation and restlessness in patients. Patients who have toxic psychosis for up to 48 h and are a danger to themselves and others may need to be admitted to the hospital for close monitoring and management.^10^ In our case, however, the patient recovered completely after the drug was stopped for 24 h. Due to laboratory limitations, the serum level of hyoscine was not measured.

Poonai et al. randomly assigned children aged 8–17 years with non‐specific colicky abdomen pain who reported to the pediatric emergency department at London Health Sciences Centre in London, Ontario, to receive hyoscine butyl bromide, 10 mg orally, or acetaminophen, 15 mg/kg orally (maximum 975 mg). In this study, hyoscine butyl bromide did not outperform acetaminophen. Of 116 individuals, 32 (27.6 percent) in the hyoscine butylbromide group experienced adverse reactions in the emergency department. Nausea, vomiting, dizziness, and photosensitivity were common adverse effects. There were no significant side effects.^11^

Anticholinergic‐induced agitation, delirium, and hallucinations are treated with physostigmine, an acetylcholinesterase inhibitor.^12^, ^13^ While the patient is being observed, a test dose of physostigmine, usually 0.5 or 1 mg, can be given intramuscularly, subcutaneously, or intravenously.^14^, ^15^ Arrhythmias or hypotension caused by the physostigmine‐induced cholinergic crisis should be monitored closely. Before prescribing physostigmine, it is important to rule out any potential contraindications such as renal hypertension, hyperthyroidism, diabetes, or coronary artery disease.^16^ Although our patient also had anticholinergic‐induced delirium and hallucinations, she was not treated with physostigmine as we preferred to withdraw our patient from the offending drug.

## 4 CONCLUSION

The safety of hyoscine in children has not yet been determined, and the effects of this medicine may be cumulative; so, the drug's usage in pediatric patients is not suggested.^16^ The take‐home lesson from this case is that clinical practitioners should be aware of common drugs having anticholinergic effects and that drug‐induced psychosis should be evaluated in young patients exhibiting acute behavioral abnormalities.

## AUTHOR CONTRIBUTIONS

NL, DN, LSJ, and UP wrote the initial draft of the manuscript. NL and DN edited the draft and reshaped it into this manuscript. The final version of the manuscript was approved by all authors and agree to be responsible for all aspects of the work.

## CONFLICT OF INTEREST

None.

### CONSENT

Written informed consent was obtained from the patient's party to publish this report in accordance with the journal's patient consent policy.

## Data Availability

The data that support the findings of this study are openly available in the Authorea repository at http://doi.org/10.22541/au.164873486.61008972/v1.
